# Evaluating the Impact of the Healthy Kids Community Challenge (HKCC) on Physical Activity of Older Youth

**DOI:** 10.3390/ijerph18063083

**Published:** 2021-03-17

**Authors:** Scott T. Leatherdale, Kathleen E. Burns, Wei Qian, Guy Faulkner, Valerie Carson

**Affiliations:** 1 School of Public Health and Health Systems, University of Waterloo, Waterloo, ON N2L 3G1, Canada; katie.burns@uwaterloo.ca (K.E.B.); wei.qian@uwaterloo.ca (W.Q.); 2 School of Kinesiology, University of British Columbia, Vancouver, BC V6T 1Z4, Canada; guy.faulkner@ubc.ca; 3 Faculty of Kinesiology, Sport, and Recreation, University of Alberta, Edmonton, AB T6G 2R3, Canada; vlcarson@ualberta.ca

**Keywords:** physical activity, youth, community-based intervention, natural experiment, longitudinal

## Abstract

(1) Background: The Healthy Kids Community Challenge (HKCC) was a community-based obesity prevention intervention funded by the Government of Ontario (Canada). (2) Methods: A quasi-experimental design was used to examine the impact of the HKCC on physical activity (PA) outcomes using both repeat cross-sectional (T1 2014–2015, n = 31,548; T2 2015–2016, n = 31,457; and T3 2016–2017, n = 30,454) and longitudinal data (n = 3906) from the COMPASS study. Grade 9–12 students in HKCC communities were placed into one of three intervention groups [T2 data collection post-HKCC finishing (IG1), T2 data collection during HKCC (IG2), and T2 data collection pre-HKCC starting (IG3)], Ontario students in non-HKCC communities were Control Group 1 (CG1) and Alberta students were Control Group 2 (CG2). (3) Results: Repeat cross-sectional results show over time the HKCC had no significant impact on PA outcomes in any of the intervention groups. Longitudinal results show a significant decrease in time spent in moderate-to-vigorous PA (IG2: −3.15 min/day) between T1 and T3 in IG2. (4) Conclusions: These results suggest the HKCC did not have an impact on improving PA outcomes among older youth in HKCC communities. Moving forward, there is a need to provide effective and sustainable interventions to promote PA among older youth.

## 1. Introduction

The 2009–2011 Canadian Measures Health Survey suggests nearly one-third of Canadian youth were classified as either overweight or obese [1]. This is a concern as youth obesity tends to persist later into adulthood [2] and can contribute to the development of numerous negative health outcomes [3]. Previous research has shown physical activity has an inverse dose–response relationship with youth overweight and obesity [4,5]. Considering the low levels of physical activity levels observed among Canadian youth populations since 2005 (fewer than 10% of Canadian youth accumulate 60 min of physical activity on at least six out of seven days per week) [6], and the inherent benefits of physical activity irrespective of the positive impact on weight outcomes [7], initiatives focused on promoting physically active lifestyles among youth are a public health priority.

In response, the Ministry of Health and Long Term Care (MOHLTC) in Ontario (Canada) announced the launch of a new provincial program, the Healthy Kids Community Challenge (HKCC) [8,9]. The HKCC, based on the EPODE (“Ensemble Prévenons l’Obésité Des Enfants”, Together Let’s Prevent Childhood Obesity) methodology [10], was a community-based, multi-sectoral program with the goal of reducing overweight and obesity among infants and children (ages 0–12 years). In brief, 45 communities across Ontario were selected to take part in the HKCC, where each selected community was supported by the MOHLTC in developing local policies and programs aimed at preventing childhood obesity. The HKCC was based around four themes (physical activity, water consumption, fruit and vegetable consumption, and screen time) that are rolled out individually in nine-month waves. The first nine-month theme of the HKCC was launched in September 2015 through a mix of active play, active transportation, and sports and structured physical activity programs and policies in each community. Public Health Ontario (PHO) is the provincial organization tasked by the Government of Ontario to perform the process implementation evaluation of the HKCC [11,12]; details of the specific intervention activities that occurred within each community for this theme are not publicly available, although some communities have presented implementation details online (e.g., Peterborough [13]). As such, despite being unable to determine if the HKCC was implemented as intended in each participating community, considering the HKCC was intended to create sustainable and effective broader community environments to promote physical activity [12] and ~$1.5 million was available for each community to take action [9], it remains important to evaluate if the initiative as a whole had any meaningful impact on youth in the participating communities relative to youth not exposed to the intervention.

PHO is also leading the outcome evaluation of the HKCC for the Province of Ontario [14]. While PHO reports that it intends to use repeat cross-sectional data from 11- and 12-year-old children collected biennially as part of the Ontario Student Drug Use and Health Survey (OSDUHS) [14] for their evaluation, the results have not yet been published or made publicly available when this was written (as of 12 March 2021). While older youth (13–18 years of age) were not part of the HKCC target population (despite evidence they are at greater risk for obesity and physical inactivity relative to their younger peers) [6,15], evaluating the potential for any spillover effects or ripple effects [16] of the HKCC, especially among these at-risk youth who are close in age to the target population, provides the opportunity to assess broader intervention impact (i.e., value) and credibility [17]. As such, the present study evaluates if the HKCC Theme 1 (Run. Jump. Play. Every Day) had an impact on physical activity outcomes among secondary school-aged youth over time within the context of a natural experiment using data available in the COMPASS Study [18,19].

## 2. Materials and Methods

The COMPASS Study (COMPASS) is a prospective cohort study (2012–2021) designed to collect hierarchical and longitudinal data from a convenience sample of Grade 9–12 students and the secondary schools they attend in Ontario and Alberta, Canada [19]. Additional details on the recruitment procedures for the convenience sample of participating schools are available online (https://uwaterloo.ca/compass-system/publications#technical; accessed on 10 March 2021). Using active-information, passive-consent parental permission protocols, eligible consenting students completed the COMPASS questionnaire (Cq) during class time (participation rate among eligible students was 78.7% in 2014–2015, 79.9% in 2015–2016, and 77.5% in 2016–2017). Students could decline to participate at any time. All procedures in the COMPASS study received ethics approval from the University of Waterloo Research Ethics Board (ORE 30118), as well as all participating school board review panels. A full description of the COMPASS host study is available online (https://uwaterloo.ca/compass-system/; accessed on 10 March 2021) and in print [19].

### 2.1. Sample

**Figure 1:**
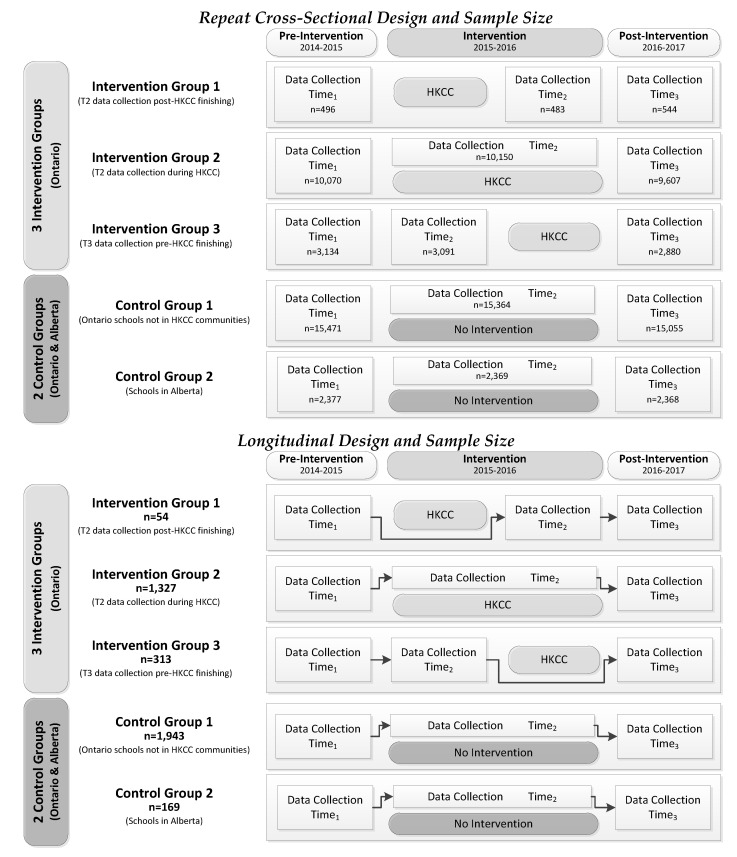
Student-level sample size for the repeat cross-sectional and longitudinal samples in the interrupted time series quasi-experimental design evaluating the impact of the Healthy Kids Community Challenge (HKCC) using data from the COMPASS study (from 2014–2015 to 2016–2017).

Data from Grade 9–12 students attending all 64 schools (57 in Ontario and 7 in Alberta) that participated in Year 3 (T1 2014–2015: baseline pre-HKCC), Year 4 (T2 2015–2016: HKCC intervention), and Year 5 (T3 2016–2017: post-HKCC follow-up) of the COMPASS host study were used. The student data from these schools were used as both repeat cross-sectional (T1 *n* = 31,548, T2 *n =* 31,457, and T3 *n =* 30,454) and longitudinal (T1–T3, *n =* 3906) samples. To determine HKCC exposure, we used the geographic location of the Ontario schools and mapped them onto the geographical boundaries for each HKCC community to determine which COMPASS schools were located within a HKCC intervention boundary and hence students attending those schools were living in a community exposed to the HKCC intervention; students attending Alberta schools would not have been exposed to the HKCC, but provided data following the same protocols and measures as Ontario students, making the Alberta data robust for use as an unexposed control group. Second, for the Ontario schools located within a HKCC community, we then used the schools’ T2 data collection date to determine if the second wave of data were collected before, during, or after the HKCC Theme 1 intervention activities specific to that HKCC community. Using this information, the 64 participating schools were divided into three intervention groups [Intervention Group 1 (IG1): T2 data collection post-HKCC finishing (*N =* 2); Intervention Group 2 (IG2): T2 data collection during HKCC (*N =* 19); and Intervention Group 3 (IG3): T2 data collection pre-HKCC starting (*N =* 7)] and two control groups [Control Group 1 (CG1): Ontario schools not in HKCC communities (*N =* 29); and Control Group 2 (CG2): Alberta schools (*N =* 7)] based on their potential exposure to the HKCC. Figure 1 presents the repeat cross-sectional and longitudinal student-level sample sizes by for the intervention and control groups.

### 2.2. Measures

In the Cq, students were asked: (a) how many minutes of moderate intensity physical activity they engaged in on each of the last seven days [in hours (0–4) and minutes (0, 15, 30, or 45)]; (b) how many minutes of hard intensity physical activity they engaged in on each of the last seven days [in hours (0–4) and minutes (0, 15, 30, or 45)]; and, (c) on how many days in the last seven days they performed exercises to strengthen or tone your muscles (e.g., push-ups, sit-ups, weight training) [0–7 days]. To calculate moderate-to-vigorous physical activity (MVPA), the scores for moderate and hard physical activities from each day were summed and divided by 7 to create an average minutes of MVPA/day score. The self-reported measures for MVPA in the Cq have an intraclass correlation coefficient of 0.75 for 1-week test–retest and 0.25 when compared to accelerometer-measured PA [20] that are comparable to self-report PA measures validated with accelerometers [21]. Consistent with the recommendations for surveillance of the Canadian 24-Hour Movement Guidelines (24-MG) for Children and Youth [22], when these data were collected, students are classified as meeting the MVPA recommendation if they had performed on average 60 min of MVPA daily and vigorous-intensity (hard) PA or strengthening activities on at least three of the last seven days, otherwise they were classified as not meeting the recommendation. Strength training (ST) was the number of days in the past week where student reported doing exercises to strengthen or tone muscles. Students also reported their gender (male, female).

### 2.3. Analyses

Descriptive statistics examined MVPA, 24-MG, and ST for the repeat cross-sectional and longitudinal samples across the three intervention (IG1, IG2, and IG3) and two control groups (CG1 and CG2). The impact of the HKCC was modeled as an interrupted time series quasi-experimental design (as shown in Figure 1) using both repeat cross-sectional and longitudinal samples where CG1 (Ontario schools not in HKCC communities) was always considered the reference group. The repeat cross-sectional data were modeled using difference-in-difference (DID) models to examine the impact of IG1, IG2, IG3, and CG2 on MVPA and ST, and a relative risk ratio model was used to examine the impact of IG1, IG2, IG3, and CG2 on meeting the 24-MG. Using the longitudinal data, we examined the impact of IG1, IG2, IG3, and CG2 on: (a) MVPA using a longitudinal linear regression model; (b) ST using a longitudinal ordinal regression model; and (c) 24-MG using a longitudinal logistic regression model. All three of the longitudinal models adjusted for within-school clustering (to account for the hierarchical nature of the data where students are clustered within schools) and gender. The benefits of the experimental design used in this study for reducing bias in the sample (i.e., DID models for the repeat cross-sectional data and longitudinal regression models adjusting for school-level variance in the longitudinal data), are described elsewhere [18]. All statistics were performed using SAS 9.4 software [23].

## 3. Results

As shown in Table 1, in the T1 cross-sectional sample, the mean MVPA was 123.5 (±91.9) min/day, the mean days of ST was 2.8 (±2.3) days/week, and the prevalence of students meeting the 24-MG was 48.4%. In the T1 longitudinal sample, the mean MVPA was 123.8 (±82.6) min/day, the mean days of ST was 2.9 (±2.2) days/week, and the prevalence of students meeting the 24-MG was 50.3%. While the T1 differences across groups for MVPA, ST, and meeting the 24-MG are all significant (*p* < 0.05), given the large sample sizes, these very modest differences being significant are not surprising. For instance, in the repeat cross-sectional sample, MVPA ranged from 122.1 min/day in IG2 to 128.7 min/day in CG2 (difference of 6.6 min/day) and strength training ranged from 2.5 days/week in IG1 to 3.0 days/week in IG3 (difference of 0.5 days/week). By T3 in the cross-sectional sample, the mean MVPA increased by 1.9 min/day (1.5% relative increase), the mean days of strength training decreased by 0.2 days/week (3.6% relative decrease), and the prevalence of students meeting the 24-MG increased 2.1% (4.3% relative increase). By T3 in the longitudinal sample, the mean MVPA decreased by 11.3 min/day (9.1% relative decrease), the mean days of ST decreased by 0.4 days/week (13.8% relative decrease), and the prevalence of students meeting the 24-MG decreased 1.9% (3.9% relative decrease).

**Figure 2:**
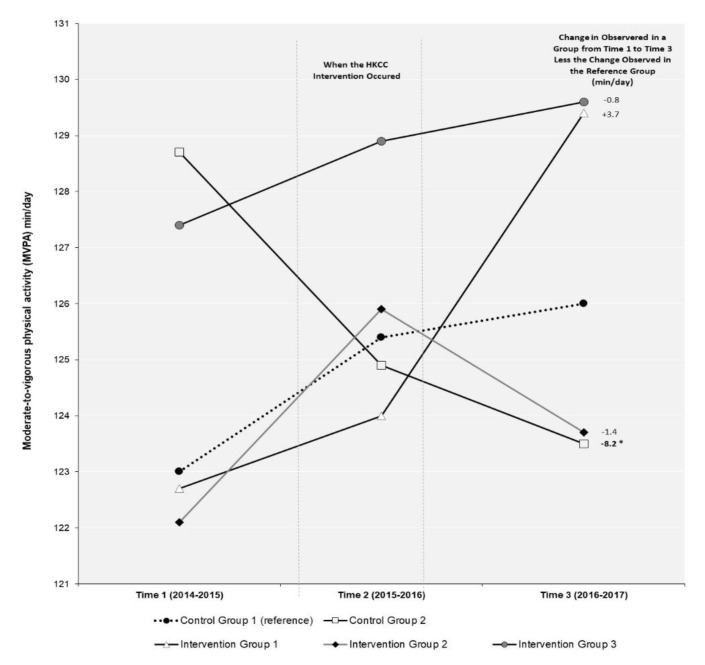
Difference-in-difference (DID) evaluation of the impact of the Healthy Kids Community Challenge (HKCC) on moderate-to-vigorous physical activity (MVPA) over time in a repeat cross-sectional sample of students in the COMPASS study (2014–2015 to 2016–2017). DID measures the differential effect of one or more groups relative to a “reference group” in a natural experiment. In a DID analysis, the effect of group membership on the outcome can be calculated by comparing the mean change over time in the outcome variable for a particular group (i.e., intervention or extra control group) compared to the change observed over time for the outcome variable in the reference group. The DID value represents the additional change in an outcome observed in that group above or below the change that occurred in the reference group.

**Figure 3:**
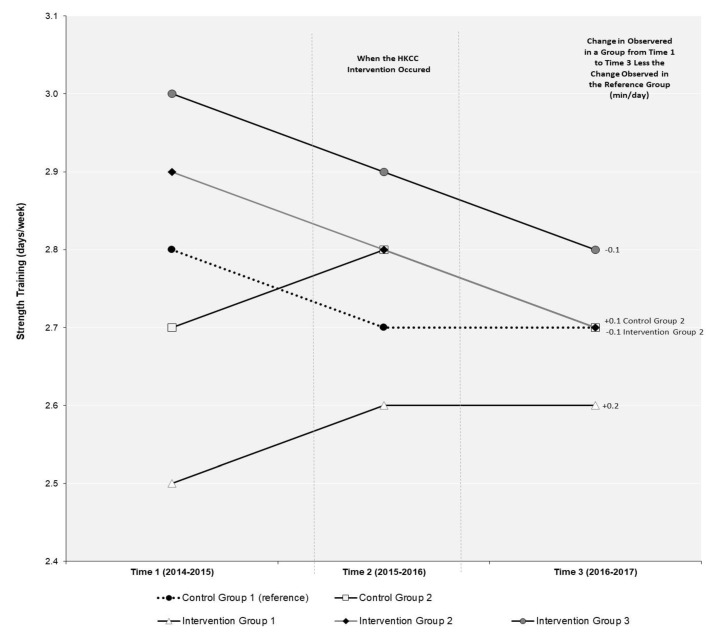
Difference-in-difference (DID) evaluation of the impact of the Healthy Kids Community Challenge (HKCC) on strength training over time in a repeat cross-sectional sample of students in the COMPASS study (2014–2015 to 2016–2017). DID measures the differential effect of one or more groups relative to a “reference group” in a natural experiment. In a DID analysis, the effect of group membership on the outcome can be calculated by comparing the mean change over time in the outcome variable for a particular group (i.e., intervention or extra control group) compared to the change observed over time for the outcome variable in the reference group. The DID value represents the additional change in an outcome observed in that group above or below the change that occurred in the reference group.

The results of the DID model for the repeat cross-sectional sample examining changes in MVPA over time are shown in Figure 2, while the results for the DID model examining changes in ST over time are in shown Figure 3. Table 2 presents the results of the relative risk ratio model examining if students report meeting the 24-MG within the repeat cross-sectional sample. Table 3 presents the results from the longitudinal sample for the longitudinal regression models examining changes in MVPA, ST, and 24-MG over time.

### 3.1. Moderate-to-Vigorous Physical Activity (MVPA)

As shown in Figure 2, the only significant change in MVPA between T1 and T3 in the repeat cross-sectional sample was in CG2. In CG2, MVPA declined by 8.2 min/day more than the change in MVPA observed in the reference group CG1. None of the changes observed in MVPA over time in the three HKCC intervention groups (IG1, IG2, and IG3) were significant in the DID model. The results of the longitudinal linear regression model (Table 3) identify students in IG2 (β-3.15, 95%CI −6.19 to −0.11) and CG2 (β-8.70, 95%CI −15.57 to −1.83) had significantly lower MVPA between T1 and T3 compared to students in the reference group CG1. There were no significant differences in MVPA over time identified for students in IG1 or IG3.

### 3.2. Strength Training (ST)

As shown in Figure 3, none of the changes in ST between T1 and T3 in the repeat cross-sectional sample were significant for the three HKCC intervention groups or CG2 in the DID model. The results of the longitudinal ordinal regression model (Table 3) also identify there were no significant differences in ST from T1 to T3 for students in the three intervention groups (IG1, IG2, and IG3) or CG2 when compared to students in the reference group CG1.

### 3.3. Meeting the 24-Hour Movement Guidelines (24-MG) 

As shown in Table 2, the relative risk of meeting the 24-MG between T1 and T3 was not significant for the three HKCC intervention groups (IG1, IG2, and IG3) or CG2 relative to the reference group CG1 in the repeat cross-sectional sample. The results of the longitudinal logistic regression model (Table 3) identify students in CG2 (OR 0.73, 95% CI 0.60–0.89) were significantly less likely to meet the 24-MG over time compared to students in the reference group CG1. There were no significant differences in the likelihood of meeting the 24-MG over time for students in the three intervention groups (IG1, IG2, and IG3) when compared to students in the reference group CG1.

## 4. Discussion

Although the HKCC was formulated to target obesity within infants and children (ages 0–12 years), assessing outcomes among older adolescents serves as a vital point of consideration when assessing the real-world impact (or spillover effect) of this large well-funded community-based provincial program. Our results suggest that, when examined using both repeat cross-sectional data and longitudinal data, there was no spillover effect of the HKCC Theme 1 (Run. Jump. Play. Every Day) intervention on improving physical activity outcomes among older youth (ages 13–18) in HKCC communities relative to older youth in control communities over time. Although research suggests even modest increases in leisure time physical activity can produce substantial health benefits [7] and economic models suggest even modestly successful community-based physical activity interventions can be a very cost-effective use of resources [24], our results are consistent with systematic reviews suggesting that, on their own, community-based interventions often have limited to no impact on youth physical activity [25,26]. It is also consistent with a systematic review of physical activity controlled trials suggesting physical activity interventions often have a negligible effect on increasing physical activity among youth populations [27]. Moving forward, it will be important to compare our results with any evaluation results eventually produced by PHO with the HKCC target population.

Conceptually, there are a few aspects of the HKCC Theme 1 intervention that may help to explain the generally null results we observed. First, a recent systematic review suggests community-based physical activity interventions are often more effective if they have broader population reach (i.e., do not just target infants and children but also youth and adult populations) [28]. Available evidence from the HKCC Program Logic Model [12] suggests HKCC communities were encouraged to implement action plans within their community that should also spill over to populations other than infants and children (e.g., social marketing and build environment resources), but it is not clear to what extent this occurred. While one study of the HKCC suggested the intervention enhanced access to health promoting programs (as described by local steering committee members) [29], evidence of actual behavior change of youth in the participating communities was not reported. Second, community-based interventions that include a robust school-based intervention component have been shown to be effective at increasing physical activity [24] and reducing weight status [25]. The HKCC Program Logic Model [12] does not appear to outline how HKCC communities would be supported to coordinate and integrate their local prevention efforts within local school-based programming, despite the EPODE program recommending the intervention success can improve when the intervention mobilizes at both the community-level and within schools [10]. Third, while EPODE also reports how successful interventions require a focus on political commitment and policy action at higher levels of government (provincial and federal) [10], these actions do not seem to be included in the HKCC Program Logic Model activities [12]. Given these limitations with the HKCC conceptually, our evaluation results may not be that unexpected. 

One of the few positive outcomes we identified was in our repeat cross-sectional analysis where student in IG1 reported an increase of 3.7 min/day of MVPA from T1 to T3. This finding is consistent with previous research suggesting that, even when effective, physical activity interventions targeting youth tend to result in a negligible increase in activity (~4 min more per day) [27]. It has also been previously suggested that community-based interventions to promote physical activity in youth populations tend to be ineffective as participating youth often do not have sufficient exposure to the actual intervention for it to work [30]. Unfortunately, the data available in the COMPASS study do not allow us to discern the actual level of exposure to the HCKK Theme 1 intervention at the individual-level among youth in participating communities. Moreover, data pertaining to the community specific HKCC intervention activities are not available, making it hard to discern what the potential intervention exposure may be at a community-level or what community-specific intervention components actually took place. Simply, it is not known if the HKCC intervention was implemented as intended. However, given the difficulties often associated with accurately measuring intervention exposure within an evaluation study, our difference-in-difference (DID) results provide a robust means for dealing with these unobserved measures of intervention exposure [31]. Considering the Province of Ontario previously reported that each of the 45 HKCC communities was eligible for up to $1.5 million to support the development and implementation of HKCC community action plans [9], one would hope such a substantial investment in each participating community would result in some meaningful level of program exposure among youth of all ages in those communities. However, our DID results in the repeat cross-sectional samples are generally consistent with our results from our longitudinal models, increasing the confidence in our results being suggestive of null-effects from the HKCC among older youth. 

### 4.1. Implications for Policy and Practice

Too often the impact of large community-based interventions goes unevaluated (especially when considering the impact on non-target populations), underestimating the true (and potential) impact of the intervention [17]. Considering the overall investment in the HKCC was expected to exceed 45 million (CAD) [9], evaluating the impact of this intervention using available data from COMPASS was a robust and efficient use of available resources for advancing our scientific understanding and improving government accountability for their prevention programming decisions. Researchers in other jurisdictions should consider the benefits of creating and then using embedded learning systems (such as COMPASS [18,19]), which allow for the real-world evaluation of natural experiments at no-extra cost to stakeholders.

### 4.2. Limitations

There are some limitations worth noting. The MOHLTC chose the communities participating within the HKCC initiative based on an application the communities submitted where they were scored and ranked based on evidence of community delivery capacity, community needs and their commitment to the maintenance of multi-sectoral partnership [9]. As such, the HKCC may have been subject to selection bias, as more health-conscious communities with existing partnerships with health units or other stakeholders may have been more inclined to apply in comparison to other communities that lacked the capacity to develop necessary partnerships. However, if this were the case, we likely would have also expected to see an increased likelihood of the intervention being successful. In addition, there is a chance the self-reported measures of health behaviors may have been subject to recall bias as a function of underreporting health behaviors within COMPASS student questionnaires. However, given COMPASS data are longitudinal, potential bias in the self-reported data is somewhat reduced as any under-reporting that may occur should remain consistent over time and the physical activity measures have demonstrated reliability and validity [20]. We also identified that similar to evidence from objective measures of youth physical activity levels over time [6], our self-reported levels of physical activity remained similar in our sample across the three years of data examined. Moreover, there is no reason to expect reporting bias for students attending a school within a HKCC community relative to those attending a school outside of the HKCC communities, especially since we did not specify to respondents how COMPASS surveillance activities would be evaluating the HKCC. In addition, not all 45 communities participating within the HKCC initiative could be captured within this analysis if schools in those communities were not participating in COMPASS and we did not have a measure of individual student exposure to the HKCC intervention among students in participating communities. Lastly, it is possible that this study was underpowered at the school-level as there were only seven schools in Alberta and 57 schools in Ontario.

## 5. Conclusions

These robust quasi-experimental results suggest the HKCC did not have any crossover effects improving physical activity outcomes among older youth in HKCC communities. The potential of population-level community-based initiatives such as the HKCC for having positive unintended benefits beyond the target audience may be limited. Moving forward, there is a need to provide effective and sustainable interventions to promote physical activity among older youth as the HKCC did not have any meaningful impact on this at-risk population. Moreover, this study also demonstrates the potential utility of using quasi-experimental research designs to evaluate the impact of real-world community-based prevention initiatives.

## Figures and Tables

**Table 1:** Moderate-to-vigorous physical activity, strength training, and meeting the Canadian 24-Hour Movement Guidelines over time by group in the repeat cross-sectional and longitudinal sample of students in the COMPASS study (from 2014–2015 to 2016–2017).

	Repeat Cross-Sectional Sample			Longitudinal Sample		
	Time 1 (2014–2015) *N* = 31,548	Time 2 (2015–2016) *N* = 31,457	Time 3 (2016–2017) *N* = 30,454	Time 1 (2014–2015) (*n* = 3906)	Time 2 (2015–2016) (*n* = 3906)	Time 3 (2016–2017) (*n* = 3906)
	Mean	Mean	Mean	Mean	Mean	Mean
**Moderate-to-Vigorous Physical Activity (min/day)**	123.5	125.8	125.4	123.8	120.0	112.5
Control Group 1 ^a^	123.0	125.4	126.0	121.5	121.5	113.1
Control Group 2 ^b^	128.7	124.9	123.5	123.0	107.5	98.8
Intervention Group 1 ^c^	122.7	124.0	129.4	116.8	117.2	112.6
Intervention Group 2 ^d^	122.1	125.9	123.7	128.1	120.7	114.1
Intervention Group 3 ^e^	127.4	128.9	129.6	121.2	115.3	110.2
**Strength Training (days/week)**	2.8	2.8	2.7	2.9	2.7	2.5
Control Group 1 ^a^	2.8	2.7	2.7	2.9	2.6	2.5
Control Group 2 ^b^	2.7	2.8	2.7	2.6	2.6	2.4
Intervention Group 1 ^c^	2.5	2.6	2.6	2.5	2.5	2.6
Intervention Group 2 ^d^	2.9	2.8	2.7	3.1	2.7	2.5
Intervention Group 3 ^e^	3.0	2.9	2.8	2.9	3.0	2.6
	%	%	%	%	%	%
**Meets Canadian 24-Hour Movement Guidelines (yes)**	48.4	50.4	50.5	48.7	50.3	46.8
Control Group 1 ^a^	47.9	50.3	50.3	46.4	50.7	46.2
Control Group 2 ^b^	48.4	48.2	48.4	46.3	47.3	32.5
Intervention Group 1 ^c^	48.4	48.4	51.7	50.9	54.9	42.0
Intervention Group 2 ^d^	47.9	50.4	50.4	51.8	50.2	49.0
Intervention Group 3 ^e^	52.4	53.4	53.0	50.7	49.8	50.2

^a^ Ontario schools not in HKCC communities; ^b^ Alberta schools; ^c^ Schools located in a HKCC community where the Time 2 data collection occurred after the HKCC intervention was finished; ^d^ Schools located in a HKCC community where the Time 2 data collection occurred during the HKCC intervention; ^e^ Schools located in a HKCC community where the Time 2 data collection occurred before the HKCC intervention was delivered.

**Table 2:** Evaluating the impact of the Healthy Kids Community Challenge (HKCC) on meeting the Canadian 24-Hour Movement Guidelines over time in a repeat cross-sectional sample of students in the COMPASS study (2014–2015 to 2016–2017).

	Pre-Intervention (T1)	Intervention (T1 to T2)	Post-Intervention (T1 to T3)
**Meets Canadian 24-Hour Movement Guidelines (yes)**	**%**	**Relative Risk Ratio (RRR)**	
Control Group 1 ^a^ (reference)	47.9	1.00	1.00
Control Group 2 ^b^	48.4	0.95	0.95
Intervention Group 1 ^c^	48.4	0.95	1.02
Intervention Group 2 ^d^	47.9	1.00	1.00
Intervention Group 3 ^e^	52.4	0.97	0.95

Notes: T1: 2014–2015; T2: 2015–2016; and T3: 2016–2017. RRR measures the ratio of the probability of an outcome for a particular group relative to the probability of an outcome in the reference group. RRR < 1 represents decreased risk as a function of group membership, RRR > 1 represents increased risk as a function of group membership, and a RRR = 1 represents no risk as a function of group membership. ^a^ Ontario schools not in HKCC communities; ^b^ Alberta schools; ^c^ Schools located in a HKCC community where the Time 2 data collection occurred after the HKCC intervention was finished; ^d^ Schools located in a HKCC community where the Time 2 data collection occurred during the HKCC intervention; ^e^ Schools located in a HKCC community where the Time 2 data collection occurred before the HKCC intervention was delivered; * *p* < 0.05.

**Table 3:** Evaluating the impact of the Healthy Kids Community Challenge (HKCC) on moderate-to-vigorous physical activity (MVPA), strength training, and meeting the Canadian 24-Hour movement Guidelines from Time 1 (2014–2015) to Time 3 (2016–2017) in the longitudinal sample of students in the COMPASS study.

**Model 1: Moderate-to-vigorous physical activity (min/day)**	***Longitudinal Linear Regression*** β (95% CI)
*Control Group 1 ^a^ (reference)*	*1.00*
Control Group 2 ^b^	−8.70 (−15.57, −1.83) *
Intervention Group 1 ^c^	2.75 (−9.55, 15.05)
Intervention Group 2 ^d^	−3.15 (−6.19, −0.11) *
Intervention Group 3 ^e^	−1.40 (−6.57, 3.77)
**Model 2: Strength Training (days/week)**	***Longitudinal Ordinal Regression*** OR (95% CI)
*Control Group 1 ^a^ (reference)*	*1.00*
Control Group 2 ^b^	0.08 (0.23, −0.08)
Intervention Group 1 ^c^	0.13 (0.50, −0.23)
Intervention Group 2 ^d^	0.04 (0.35, −0.27)
Intervention Group 3 ^e^	0.07 (0.31, −0.17)
**Model 3: Meets Canadian 24-Hour Movement Guidelines (%)**	***Longitudinal Logistic Regression*** OR (95% CI)
*Control Group 1 ^a^ (reference)*	*1.00*
Control Group 2 ^b^	0.73 (0.60, 0.89) *
Intervention Group 1 ^c^	0.84 (0.59, 1.19)
Intervention Group 2 ^d^	0.95 (0.86, 1.03)
Intervention Group 3 ^e^	0.99 (0.85, 1.15)

Notes: All models are adjusted for within-school clustering and gender. Model 1 (*n* = 3538), Model 2 (*n* = 3628), and Model 3 (*n* = 3538). ^a^ Ontario schools not in HKCC communities; ^b^ Alberta schools; ^c^ Schools located in a HKCC community where the Time 2 data collection occurred after the HKCC intervention was finished; ^d^ Schools located in a HKCC community where the Time 2 data collection occurred during the HKCC intervention; ^e^ Schools located in a HKCC community where the Time 2 data collection occurred before the HKCC intervention was delivered; * *p* <0.05.

## Data Availability

The data presented in this study are available on request from the corresponding author or from the COMPASS data use application online form (https://uwaterloo.ca/compass-system/sites/ca.compass-system/files/uploads/files/compass_data_use_application_2020.pdf). The data are not publicly available as the study is still ongoing.
